# Analysis of Rabies in China: Transmission Dynamics and Control

**DOI:** 10.1371/journal.pone.0020891

**Published:** 2011-07-14

**Authors:** Juan Zhang, Zhen Jin, Gui-Quan Sun, Tao Zhou, Shigui Ruan

**Affiliations:** 1 Department of Mathematics, North University of China, Taiyuan, Shan'xi, People's Republic of China; 2 Web Sciences Center, University of Electronic Science and Technology of China, Chengdu, Sichuan, People's Republic of China; 3 Department of Modern Physics, University of Science and Technology of China, Hefei, Anhui, People's Republic of China; 4 Department of Mathematics, University of Miami, Coral Gables, Florida, United States of America; Fundació Institut Germans Trias i Pujol; Universitat Autònoma de Barcelona CibeRES, Spain

## Abstract

Human rabies is one of the major public-health problems in China. The number of human rabies cases has increased dramatically in the last 15 years, partially due to the poor understanding of the transmission dynamics of rabies and the lack of effective control measures of the disease. In this article, in order to explore effective control and prevention measures we propose a deterministic model to study the transmission dynamics of rabies in China. The model consists of susceptible, exposed, infectious, and recovered subpopulations of both dogs and humans and describes the spread of rabies among dogs and from infectious dogs to humans. The model simulations agree with the human rabies data reported by the Chinese Ministry of Health. We estimate that the basic reproduction number 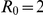 for the rabies transmission in China and predict that the number of the human rabies is decreasing but may reach another peak around 2030. We also perform some sensitivity analysis of 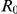 in terms of the model parameters and compare the effects of culling and immunization of dogs. Our study demonstrates that (i) reducing dog birth rate and increasing dog immunization coverage rate are the most effective methods for controlling rabies in China; and (ii) large scale culling of susceptible dogs can be replaced by immunization of them.

## Introduction

Rabies is an acute and fatal zoonotic disease. The rabies virus infects the central nervous system and causes disease in the brain. Once symptoms of the disease develop, its mortality rate is 100%. Rabies can infect animals and also can be spread to humans through the bite or scratch of an infected dog or cat [1], [2]. All species of mammals are susceptible to rabies virus infection, but dogs remain the main carrier of rabies and are responsible for most of the human rabies deaths worldwide [3]. Rabies is widely distributed around the globe. More than 55,000 people die of rabies each year. About 95% of human deaths occur in Asia and Africa [2].

Human rabies in China was first reported in about 556 BC and has persisted for more than 2500 years [4]. Since 1950, the second year after the establishment of People's Republic of China, human rabies has been classified as a class II infectious disease in the National Stationary Notifiable Communicable Diseases [5], [6], and the annual data of human rabies have been archived by the Chinese Center for Disease Control and Prevention. From 1950 to 2010, 124,255 human rabies cases were reported in China [6]–[9], an average of 2,037 cases per year. Nowadays, China is second only to India worldwide in the number of people killed by rabies every year [8].

In the last 60 years, China experienced a few major epidemics of human rabies. The first peak occurred from 1956 to 1957 with about 2,000 cases in both years, followed by substantial decreases in the early 1960s. The number of cases reached 2,000 again in 1969 and increased to the historical record of 7,037 cases in 1981. During the 1980s, more then 5,000 cases were reported annually. In the 1990s, the number of cases declined rapidly from 3,520 in 1990 to 159 in 1996 [6], [8]. Since then, the number of human rabies case has increased steadily again and reached another peak in 2007 with 3,300 cases [7], [8]. From 1996 to 2010, 24,067 human rabies cases were reported [8], [9]. Though human rabies were reported in almost all provinces in China [5], nearly 60% of the total rabies cases in China were reported in the southern Guangdong, Guangxi, Guizhou, Hunan, and Sichuan provinces [8]. It is believed that the increase of rabies deaths results from a major increase in dog ownership and a very low rate of rabies vaccination [8]. In rural areas, about 70 percent of households keep dogs and low vaccination coverage of dogs is widespread, largely because of poor awareness of rabies and the high cost of vaccination. Moveover, owned dogs usually have not been registered and the number of dogs is estimated at 80–200 millions [1].

Although the recent reemergence of human rabies in China has attracted enormous attention of many researchers, the transmission dynamics of rabies in China is still poorly understood. Zhang et al. [6] analyzed the 108,412 human rabies cases in China from 1950 to 2004. They suggested that the rabies epidemics in China may be explained by dog population dynamics, untimely and inappropriate postexposure prophylaxis (PEP) treatment, and the existence of healthy carrier dogs. Si et al. [10] examined the 22,527 human rabies cases from January 1990 to July 2007 and the details of 244 rabies patients, including their anti-rabies treatment of injuries or related incidents. They concluded that the failure to receive PEP was a major factor for the increase of human cases in China. Song et al. [7] investigated the status and characteristics of human rabies in China between 1996 and 2008 to identify the potential factors involved in the emergence of rabies.

Mathematical modeling has become an important tool in analyzing the epidemiological characteristics of infectious diseases and can provide useful control measures. Various models have been used to study different aspects of rabies [11]–[28]. Anderson et al. [11] pioneered a deterministic model consisting of three subclasses, susceptible, infectious and recovered, to explain epidemiological features of rabies in fox populations in Europe. A susceptible, exposed, infectious, and recovered (SEIR) model was proposed by Coyne et al. [12], and lately was also used by Childs et al. [13], to predict the local dynamics of rabies among raccoons in the United States. Dimitrov et al. [14] presented a model for the immune responses to a rabies virus in bats. Clayton et al. [15] considered the optimal control of an SEIRS model which describes the population dynamics of a rabies epidemic in raccoons with seasonal birth pulse. Besides these deterministic models, discrete deterministic and stochastic models (Artois et al. [18], Allen at al. [19]), continuous spatial models (Källen et al. [20]), and stochastic spatial models (Smith et al. [21], Russell et al. [22]) have also been employed to study the transmission dynamics of rabies. We refer to a review by Sterner and Smith [17] and a thesis by Beyer [23] for more detailed discussions on different rabies models.

All of the above mentioned papers were about modeling wildlife rabies, recently there have been some studies on modeling canine and human rabies. Hampson et al. [24] observed rabies epidemics cycles with a period of 3–6 years in dog populations in Africa, built a susceptible, exposed, infectious, and vaccinated model with an intervention response variable, and showed significant synchrony. Carroll et al. [25] created a continuous compartmental model to describe rabies epidemiology in dog populations and explored three control methods: vaccination, vaccination plus fertility control, and culling. Wang and Lou [26] and Yang and Lou [27] used ordinary differential equation models to characterize the transmission dynamics of rabies between humans and dogs. Zinsstag et al. [28] extended existing models on rabies transmission between dogs to include dog-to-human transmission and concluded that combining human PEP with a dog-vaccination campaign is more cost-effective in the long run.

To understand the transmission dynamics of rabies in China and to explore effective control and prevention measures, in this paper we propose a deterministic SEIRS model to describe the spread of rabies among dogs and from dogs to humans. Both dogs and humans are included and are classified into susceptible, exposed, infectious, and recovered classes. We first simulate the number of human rabies cases in China from 1996 to 2010 reported by the Chinese Ministry of Health. Numerical simulations support the data reasonably well. We then estimate that the basic reproduction number 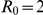 for rabies transmission in China. We also perform some sensitivity analysis of 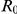 in terms of the model parameters and compare the effects of culling and immunization of dogs. Our study demonstrates that (i) reducing dog birth rate and increasing the dog immunization coverage rate are the most effective methods in controlling human rabies infection in China; and (ii) culling of dogs can be replaced by immunization of dogs.

## Methods

Both dogs and humans are considered in this study. We classify each of them into four subclasses: susceptible, exposed, infectious and recovered, with dog sizes denoted by 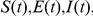 and 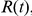 and human sizes denoted by 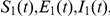 and 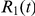, respectively.

### Mathematical Model

**Figure 1:**
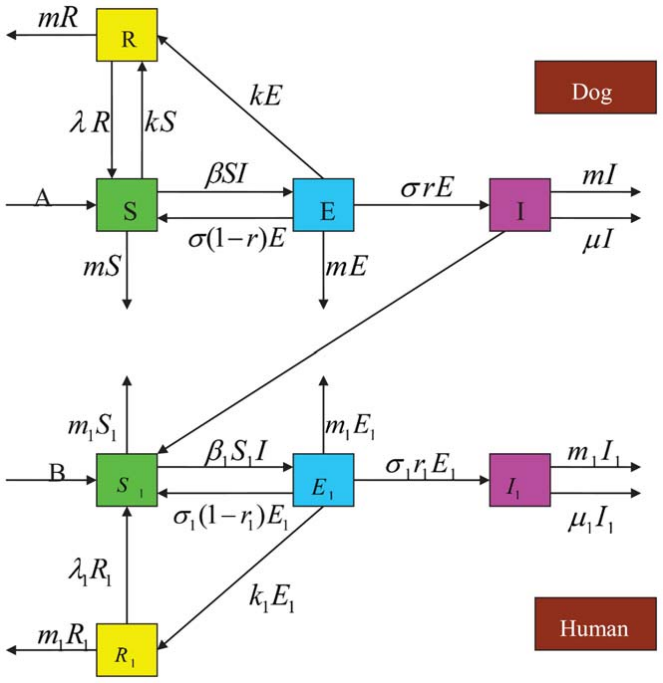
Transmission diagram of rabies among dogs and from dogs to humans. 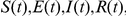
 and 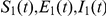 represent susceptible, exposed, infectious and recovered dogs and humans, respectively.

Our assumptions on the dynamical transmission of rabies among dogs and from dogs to humans are demonstrated in the flowchart (Fig. 1). The model is a system of eight ordinary differential equations:
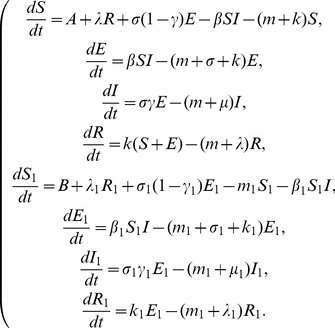
(1)


All parameters are positive. For the dog population, 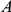 describes the annual birth rate; 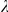 denotes the loss rate of vaccination immunity; 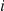 represents the incubation period of infected dogs so that 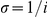 is the time duration in which infected dogs remain infectious; 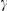 is the risk factor of clinical outcome of exposed dogs, so 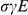 represents those exposed dogs that develop clinical rabies and 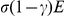 denotes those that do not develop clinical rabies and return to the susceptible class; 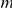 is the natural death rate; 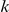 is the vaccination rate; 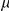 is the disease-related death rate; 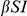 describes the transmission of rabies by interactions between infectious dogs and susceptible dogs. For the human population, 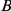 is the annual birth rate; 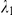 represents the loss rate of vaccination immunity; 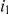 denotes the incubation period of infected individuals so 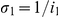 is the time duration of infectiousness of infected persons; 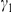 is the risk factor of clinical outcome of exposed humans, so 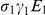 represents those exposed individuals develop into the infectious class and the rest 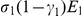 return to the susceptible class; 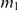 is the natural death rate; 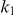 is the vaccination rate; 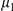 is the disease-related death rate. The term 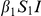 describes the transmission of rabies from infectious dogs to susceptible humans.

### Basic reproduction number and stability of equilibria

Define the basic reproduction number by (see [29], [30])
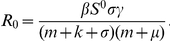



Equilibria are obtained by setting the right side of each of the eight differential equations equal to zero. If 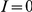, it is easy to deduce the disease-free equilibrium:
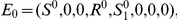
where
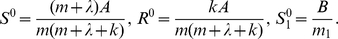



If 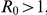 we can derive the unique endemic equilibrium:
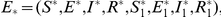
where
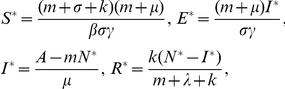


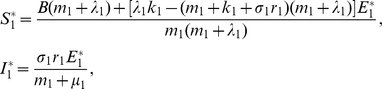


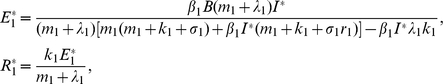
in which
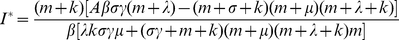


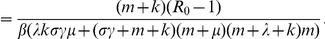



For the disease-free equilibrium point, we have the following property.

#### Theorem 1

*If* 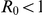, *then (a) the disease-free equilibrium* 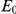 *of system (1) is locally asymptotically stable. (b) the disease-free equilibrium* 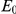 *of system (1) is globally asymptotically stable in the region* 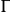.

We also have the following result on the stability of the endemic equilibrium.

#### Theorem 2

*If* 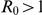, *then the endemic equilibrium* 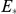 *of system (1) is locally asymptotically stable in the region* 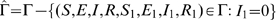. *All solutions in* 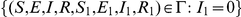 *tend toward the disease-free equilibrium* 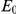.

The proofs of Theorems 1 and 2 are given in Supporting Information S1.

### Estimation of Epidemiological Parameters

In order to carry out the numerical simulations, we need to estimate the model parameters. The data concerning human rabies from 1996 to 2010 are obtained mainly from epidemiological bulletins published by the Chinese Ministry of Health [8], . However, the data involving dogs cannot be acquired easily. We have to rely on online news, our estimation or data fitting. The values of parameters are listed in Table 1. We explain the parameter values as follows: (a) The number of dogs was estimated to be 30 millions in 1996 and 75 millions in 2009 [8]. (b) The incubation period of rabies is 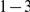 months. We select the medium value: 2 months. So 
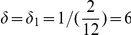
. According to the protection period of rabies vaccine, we assume that 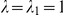. The probability of clinical outcome of the exposed is 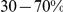. Here, we assume that it is 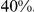 So 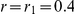. (c) The rate of vaccination is the product of efficiency and the coverage rate of rabies vaccine. Efficiency of rabies vaccine is about 90%. However, the rates of vaccine coverage for dogs and humans are low. Considering a large number of stray dogs and the poor awareness of people in rural areas, we assume that they are equal to 10% and 60%, respectively. (d) The transmission rates 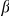 and 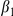 are obtained by fitting in simulations.

**Table 1:** Description of parameters in model (1).

Parameters	Value	Unit	Comments	Source
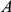	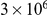	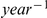	annual crop of newborn puppies	fitting
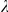	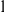	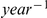	dog loss rate of vaccination immunity	assumption
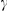	0.4	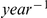	risk of clinical outcome of exposed dogs	[39]
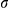	6	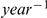	the reciprocal of the dog incubation period	assumption
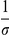	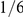	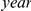	dog incubation period	assumption
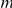	0.08	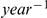	dog natural mortality rate	assumption
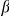	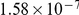	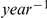	dog-to-dog transmission rate	fitting
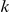	0.09	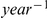	dog vaccination rate	[8]
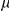	1	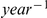	dog disease-related death rate	[8]
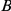	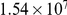	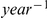	human annual birth population	[40]
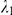	1	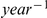	human loss of vaccination immunity	assumption
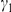	0.4	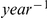	risk of clinical outcome of exposed humans	[39]
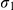	6	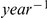	the reciprocal of the human incubation period	[39]
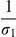	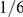	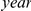	human incubation period	[39]
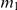	0.0066	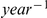	human natural mortality rate	[41]
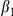	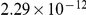	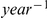	dog-to-human transmission rate	fitting
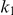	0.54	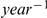	human vaccination rate	[8]
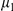	1	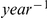	human disease-related death rate	[8]

## Results

### Numerical Simulations

**Figure 2:**
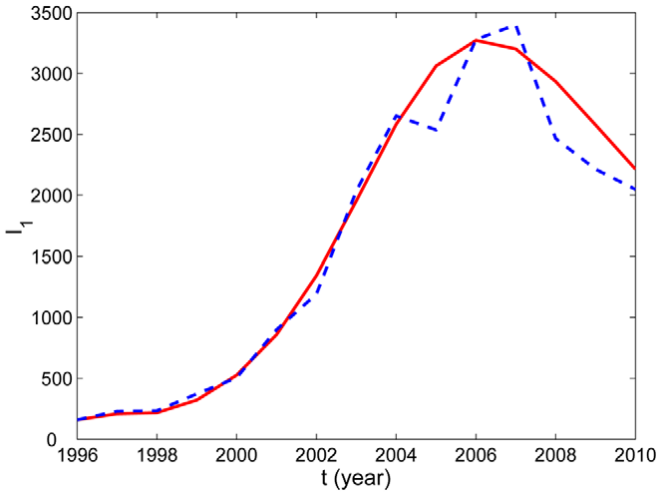
The comparison between the reported human rabies cases in mainland China from 1996 to 2010 and the simulation of 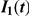 from the model. The dashed curve represents the data reported by the Chinese Ministry of Health while the solid curve is simulated by using our model. The values of parameters are given in Table 1. The initial values used in the simulations were 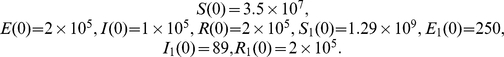.

**Figure 3:**
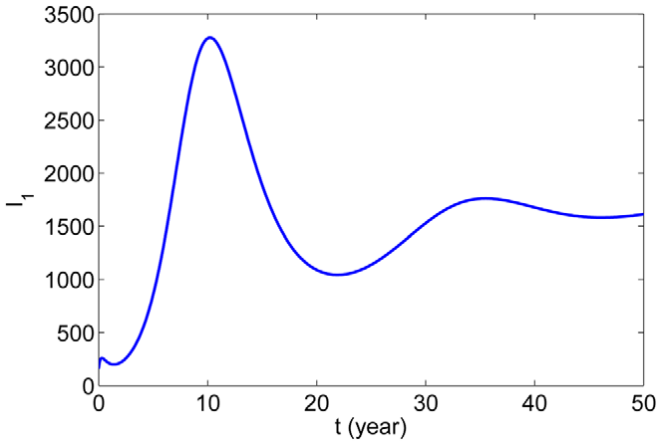
The tendency of human rabies cases 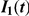 in 50 years.

The numerical simulation of human rabies cases in China from 1996 to 2010 is shown in Fig. 2, indicating that our model provides a good match to the reported data. Our model does not include culling of dogs. In 2006, 50,000 dogs were slaughtered in Yunnan Province after three people died of rabies. Thousands of stray and owned dogs were killed in response to eight cases of human rabies in Hanzhong City in 2009. The awareness of rabies for people in recent years has been enhanced gradually. This may explain why the number of human rabies cases decreased in most recent years. This demonstrates further that our model has certain rationality. Moreover, our model indicates the tendency of the rabies epidemics with time, which is presented in Fig. 3. It shows that the number of human rabies cases will decrease steadily in the next 7 or 8 years, then increase again and reach another peak (about 1750) in 2030, and finally become stable. Therefore, if no further effective prevention and control measures are taken, the disease will not vanish.

### Basic Reproductive Number for Rabies in China

Based on the parameter values given in Table 1, we estimate that the basic reproduction number 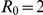 for rabies transmission in China. For rabies in Africa, Hampson et al. [31] obtained that 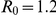 according to the data from 2002 to 2007 when the peak of animal rabies cases was less then 30 weekly, which is far less than 393 the peak of monthly human rabies cases in China. Zinsstag et al. [28] also estimated the effective reproductive ratio to be 1.01 through a research framework for rabies in an African city. Also for the rabies in USA in the 1940s when the annual reported cases varied from 42 to 113 and sharply increased in 1948, it was estimated that 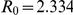 [32]. From these, it can be seen that our estimate of 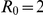 is reasonable. More discussions of 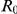 for outbreaks of rabies around the world can be found in [31], [32].

### Sensitivity Analysis

**Figure 4:**
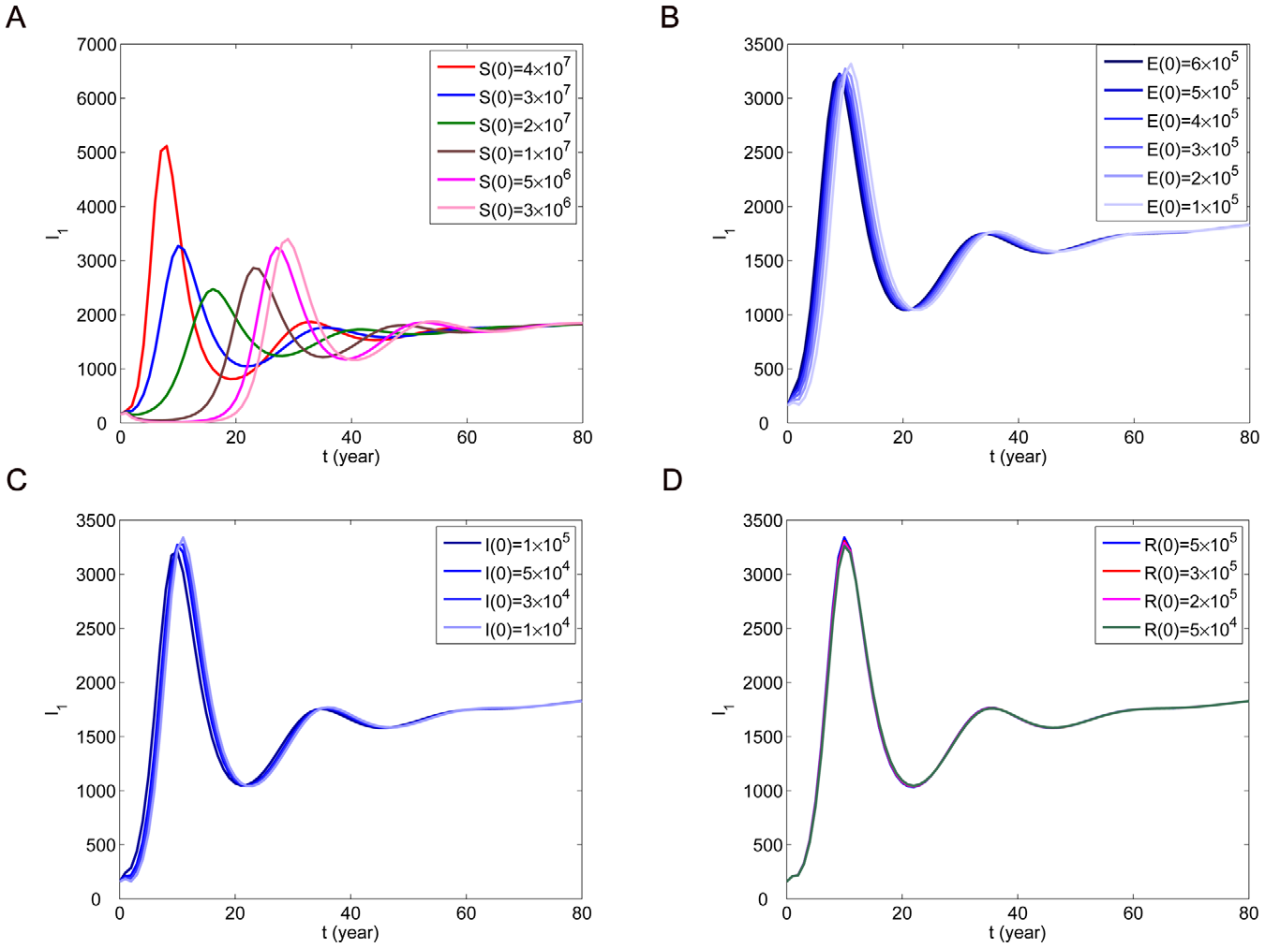
The influence of initial conditions of dogs on the number of human rabies cases 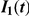. (A) 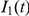 for different values of 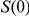. (B) 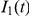 for different values of 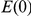. (C) 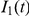 for different values of 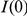. (D) 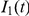 for different values of 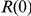.

**Figure 5:**
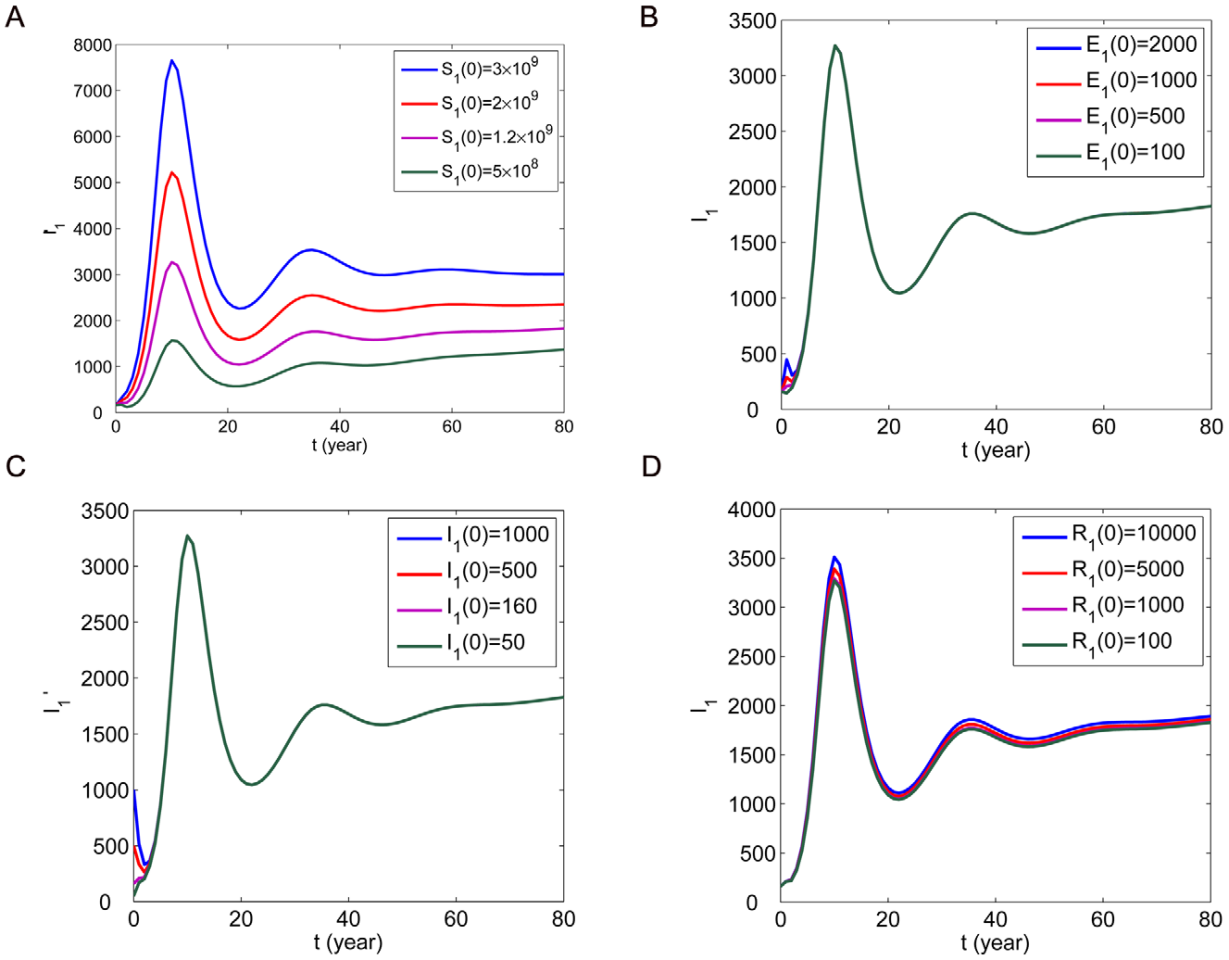
The influence of initial conditions about humans on the number of human rabies cases 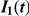. (A) 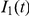 for different values of 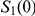. (B) 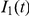 for different values of 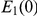. (C) 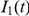 for different values of 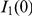. (D) 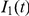 for different values of 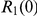.

Firstly, we look at the influence of initial conditions on the number of infected human rabies cases 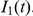 From Figs. 4 and 5, we can see that the effects of 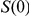 and 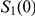 are stronger and other initial conditions have little or almost no influence on 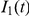. Moreover, we find that the initial conditions about dogs can influence not only the number of human rabies cases but also the time of rabies case peak. The initial conditions about humans do not have such effects. We also observe that the peak of the initial outbreak would be postponed if 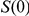 is decreasing.

**Figure 6:**
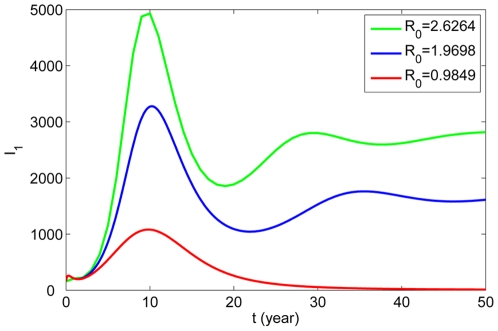
The variations of the infected human rabies cases 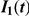 for different values of 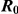. Here 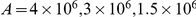, 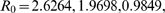 respectively, other parameters are as in Table 1.

**Figure 7:**
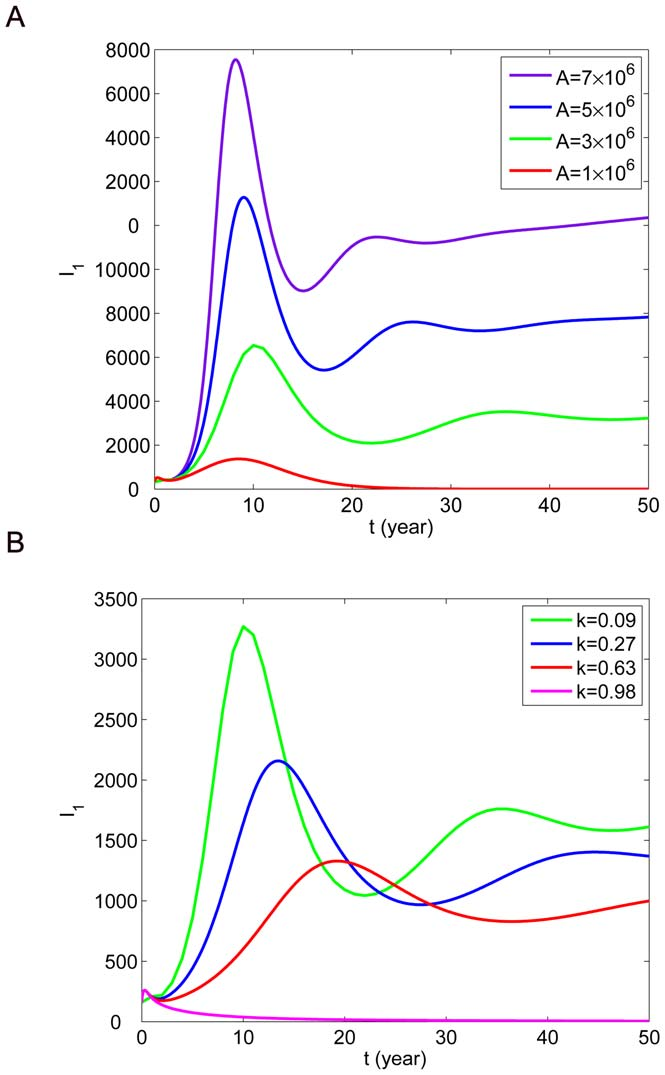
The influence of parameters 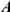 and 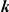 on the number of human rabies cases 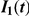. (A) 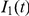 in terms of different values of 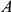. (B) 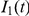 in terms of different values of 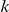.

**Figure 8:**
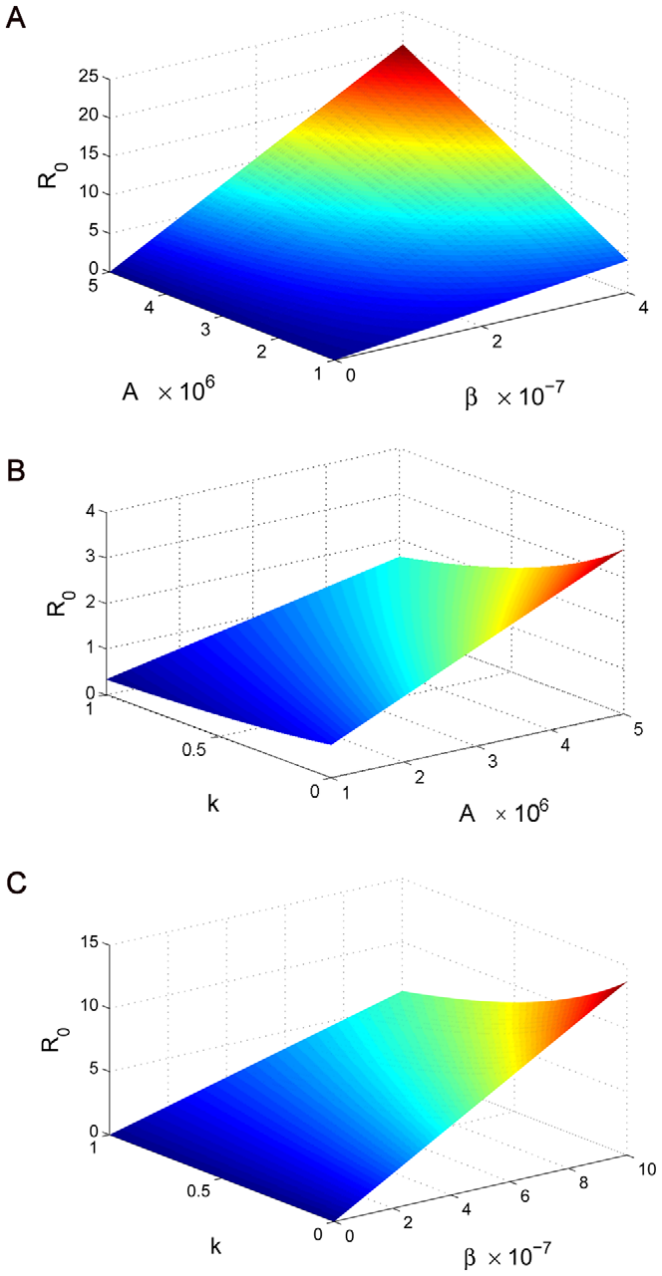
The combined influence of parameters on 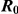. (A) 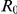 in terms of 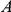 and 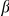. (B) 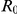 in terms of 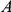 and 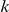. (C) 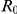 in terms of 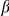 and 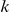.

Next, to find better control strategies for rabies infection, we perform some sensitivity analysis of 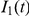 and the basic reproduction number 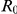 in terms of the model parameters. First, we show variations of 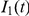 with time for different values of 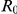 in Fig. 6. We can see that 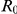 is really the threshold for the establishment of the disease in the susceptible pool and the number of infections increases with the increase of 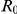. The influences of 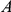 and 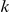 on 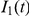 are shown in Fig. 7. It can be observed that 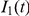 decreases as 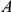 is declining or 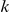 is increasing. When 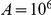 and 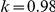, the disease can die out. Moreover, we find that the decrease of 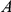 cannot delay the time of the first peak while an increase of 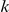 can. Furthermore, the influences of 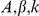 on 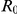 are given in Fig. 8. It is clear that 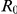 changes more quickly when both 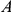 and 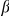 vary. When 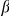 is very small, the disease can be eliminated even if 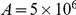. When 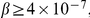 the disease cannot be eliminated even if 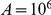. From (B) and (C) in Fig. 8, it is clear that when 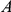 or 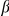 is very small, the disease can disappear even if 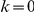. When 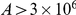 or 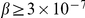, the disease cannot be eliminated even if 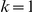. Hence, it indicates that the influence of 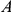 and 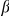 on the basic reproduction number 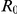 is greater. Fig. 8 reflects that whatever dog vaccination rate is, when the annual crop of newborn puppies is greater than 3 million and dog-to-dog transmission rate is greater than 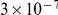, 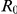 cannot be below 1. However, it is difficult to control 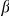.

Currently, in China the annual crop of newborn puppies can exceed 5 million and the proportion of immunized dogs is only about 10%, which is too low. According to the current incidence 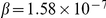, we know that if the annual crop of newborn puppies 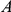
(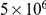) is not reduced, it is impossible to have 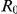 below 1; if 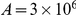, it is necessary to keep 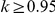; if 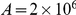, it is necessary to keep 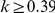.

The above analysis demonstrates that human rabies can be controlled with two strategies: reducing the annual crop of newborn puppies and increasing the dog immunization rate at the same time, which can also reduce the incidence rate 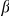.

### The Equal Effect of Culling Rate and Immunization Rate

**Figure 9:**
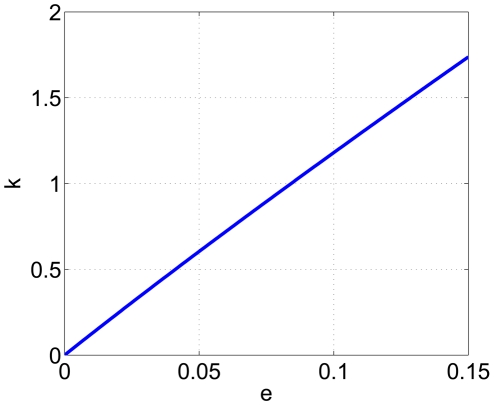
The equal effect of culling and immunization of dogs.

It has been found that in Europe culling as a means of rabies control was not effective once rabies became established within the fox population (MacDonald [33]) and had only limited success (Smith and Harris [34]). Recently, some studies suggest the strategy of culling dogs to control rabies (Kureishi et al. [35], Hu et al. [5], etc.) and some cities have in fact taken this measure. Here, we particularly discuss the influence of culling dogs. By adding the terms to describe the culling of dogs, the model becomes the following:
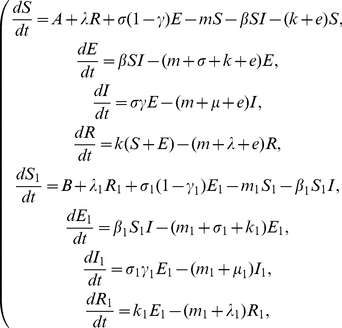
(2)where 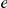 is the dog culling rate. We are interested in comparing the levels of culling and immunizing that are necessary to provide the same effect. The comparison is shown in Fig. 9. It demonstrates that the culling rate must be about 10 times the immunization rate to have an equal effect. This indicates that, under the same condition, immunizing 1% of the susceptible and exposed dogs has the same effect as culling about 12.38% of dogs. Culling of infected dogs is necessary in controlling the outbreak for a short term as suggested by some studies, our results show that large scale culling of susceptible dogs can be replaced by immunization of them.

## Discussion

Rabies is one of the biggest public health threats in China. Facing up to the epidemic situation in China, both the central and local governments have been seeking forceful methods to reduce rabies transmission. Various prevention and control measures have been proposed by many researchers which include: (i) strengthening the postexposure prophylaxis (PEP) schedules delivered to rabies patients [7], [10]; (ii) culling of dogs, in particular stray dogs [35]; (iii) increasing the vaccination coverage in dogs [36]. Some researches suggest that combining these methods can be more effective in controlling the rabies. For example, Hu et al. [5] came up with strategies to control and prevent human rabies that include public education and awareness about rabies, pet vaccination programs, culling of stray animals, and enhancing PEP for infected patients. However, the large-scale culling of dogs, criticized by pet owners and animal protection activists, is controversial and there is a lack of evidence of its effectiveness in controlling dog population or rabies (WHO [37]). In fact, culling may remove vaccinated dogs, increase immigration, disrupt social organization, and lose public support, which make rabies control more difficult (Carroll et al. [25]).

In this article, in order to explore effective control and prevention measures we proposed a susceptible, exposed, infectious, and recovered model to study the transmission dynamics of rabies in China. The model describes the transmission of rabies among dogs and from dogs to humans. The model simulations agreed with the human rabies data reported by the Chinese Ministry of Health and gave an estimate of the basic reproduction number 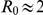. The sensitivity analysis of 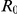 in terms of the model parameters and the comparison of the effects of culling and immunization of dogs demonstrate that (i) controlling dog birth rate and increasing dog immunization coverage rate are the most effective methods for controlling rabies in China; and (ii) large scale culling of susceptible dogs can be replaced by immunization of them.

The characteristics of rabies epidemics in China include the large size of the dog population, the extremely low dog vaccination rate, poor understanding of the transmission dynamics of rabies, inadequate treatment of the infected patients, and the countrywide scale of the disease [8]. WHO recommends that 70% of dogs in a population should be immunized in order to eliminate or prevent outbreaks of rabies. However, the dog immunization rate in China has yet reached 10%, which is even lower in the countryside [8]. So increasing dog vaccination coverage rate is necessary and crucial in control rabies transmission in China. We suggest decreasing the dog birth rate in order to reduce the dog population and stop culling dogs and try to vaccinate these dogs instead. For pet dogs, registration and immunization should be mandatory; the price of vaccine should be reduced; the awareness of prevention rabies for dog owners should be enhanced; and contraception measures should be taken. For stray dogs, food baits containing oral vaccine or abortifacient in capsules could be distributed (Roberts and Aubert [38]) and the fostering of stray dogs could be introduced and encouraged.

## Supporting Information

Supporting Information S1 Stability of the disease-free and endemic equilibria is given in this file.
(PDF)

## References

[1] Tang XC, Luo M, Zhang SY, Anthony RF, Hu RL (2005). Pivotal role of dogs in rabies transmission, China.. Emerg Inf Dis.

[2] WHO (2010). Rabies.

[3] CDC (2010). Rabies.

[4] Wang XJ, Huang JT, Yu YX (2001). Epidemiology.. Rabies and Rabies Vaccine.

[5] Hu RL, Tang Q, Tang JR, Fooks AR (2009). Rabies in China: An Update.. Vector-borne and Zoonotic Disease.

[6] Zhang YZ, Xiong CL, Xiao DL, Jiang RJ, Wang ZX (2005). Human Rabies in China.. Emerg Infect Dis.

[7] Song M, Tang Q, Wang DM, Mo ZJ, Guo SH (2009). Epidemiological investigations of human rabies in China.. BMC Infect Dis.

[8] Ministry of Health of the People's Republic of China (2009). The Status of Prevention and Control of Rabies in China. (zhongguo kuangquanbing fangzhi xiankuang).

[9] Ministry of Health of the People's Republic of China, Bulletins (2010). http://www.moh.gov.cn/publicfiles/business/htmlfiles/mohbgt/pwsbgb/index.htm.

[10] Si H, Guo ZM, Hao YT, Liu YG, Zhang DM (2008). Rabies trend in China (1990-2007) and post-exposure prophylaxis in the Guangdong Province.. BMC Infect Dis.

[11] Anderson RM, Jackson HC, May RM, Smith AM (1981). Population dynamics of fox rabies in Europe.. Nature.

[12] Coyne MJ, Smith G, McAllister FE (1989). Mathematic model for the population biology of rabies in raccoons in the mid-Atlantic states.. Am J Vet Res.

[13] Childs JE, Curns AT, Dey ME, Real LA, Feinstein L (2000). Predicting the local dynamics of epizootic rabies among raccoons in the United States.. Proc Natl Acad Sci USA.

[14] Dimitrov DT, Hallam TG, Rupprecht CE, Turmelle AS, McCracken GF (2007). Integrative models of bat rabies immunology, epizootiology and disease demography.. J Theor Biol.

[15] Clayton T, Duke-Sylvester S, Gross LJ, Lenhart S, Real LA (2010). Optimal control of a rabies epidemic model with a birth pulse.. J Biol Dynam.

[16] Smith GC, Cheeseman CL (2002). A mathematical model for the control of diseases in wildlife populations: culling, vaccination and fertility control.. Ecol Model.

[17] Sterner RT, Smith GC (2006). Modelling wildlife rabies: Transmission, economics, and conservation.. Biol Conservat.

[18] Artois M, Langlais M, Suppo C (1997). Simulation of rabies control within an increasing fox population.. Ecol Model.

[19] Allen LJS, Flores DA, Ratnayake RK, Herbold JR (2002). Discrete-time deterministic and stochastic models for the spread of rabies.. Appl Math Comput.

[20] Kallen A, Arcuri P, Murray JD (1985). A simple model for the spatial spread and control of rabies.. J Theor Biol.

[21] Smith DL, Lucey B, Waller LA, Childs JE, Real LA (2002). Predicting the spatial dynamics of rabies epidemic on heterogeneous landscapes.. Proc Natl Acad Sci USA.

[22] Russell CA, Real LA, Smith DL (2006). Spatial control of rabies on heterogeneous landscapes.. PLoS ONE.

[23] Beyer HB (2010). Epidemiological Models of Rabies in Domestic Dogs: Dynamics and Control..

[24] Hampson K, Dushoff J, Bingham J, Bruckner G, Ali YH (2007). Synchronous cycles of domestic dog rabies in Sub-Saharan Africa and the impact of control effort.. Proc Natl Acad Sci USA.

[25] Carroll MJ, Singer A, Smith GC, Cowan DP, Massei G (2010). The use of immunocontraception to improve rabies eradication in urban dog populations.. Wildlife Research.

[26] Wang X, Lou J (2008). Two dynamic models about rabies between dogs and human.. J Biol Syst.

[27] Yang W, Lou J (2009). The dynamics of an interactional model of rabies transmitted between human and dogs.. Bollettino U M I.

[28] Zinsstag J, Durr S, Penny MA, Mindekem R, Roth F (2009). Transmission dynamic and economics of rabies control in dogs and humans in an African city.. Proc Natl Acad Sci USA.

[29] van den Driessche P, Watmough J (2002). Reproduction numbers and sub-threshold endemic equilibria for compartmental models of disease transmission.. Math Biosci.

[30] Diekmann O, Heesterbeek JAP, Roberts MG (2010). The construction of next-generation matrices for compartmental epidemic models.. J Royal Soc Interface.

[31] Hampson K, Dushoff J, Cleaveland S, Haydon DT, Kaare M (2009). Transmission dynamics and prospects for the elimination of canine rabies.. PLoS Biol.

[32] Coleman PG, Dye C (1996). Immunization coverage required to prevent outbreaks of dog rabies.. Vaccine.

[33] MacDonald DW (1980). Rabies and Wildlife: A Biologist's Perspective.

[34] Smith GC, Harris S, Putman RJ (1989). The control of rabies in urban fox populations.. Mammals as Pests.

[35] Kureishi A, Xu LZ, Stiver HG (1992). Rabies in China: Recommendations for control.. Bull WHO.

[36] Wu XF, Hu RL, Zhang YZ, Dong GM, Rupprecht CE (2009). Reemerging rabies and lack of systemic surveillance in People's Republic of China.. Emerg Inf Dis.

[37] WHO (2004). WHO Expert Consultation on Rabies: First Report..

[38] Roberts MG, Aubert MFA (1995). A model for the control of Echinococcus multilocularis in France.. Vet Parasitol.

[39] Baidu Baike (2011). Rabies.. http://baike.baidu.com/view/10630.htm.

[40] Fxxue (2011). Chinese Statistics on Annual Birth Population.. http://fxxue.blog.hexun.com/44596551_d.html.

[41] National Bureau of Statistics of China (2009). China Demographic Yearbook of 2009.. http://www.stats.gov.cn/tjsj/ndsj/2009/indexch.htm.

