# Supplementary material for: Analysis of Rabies in China: Transmission Dynamics and Control
Source: PLoS One. 2011 Jul 14;6(7):e20891. doi: 10.1371/journal.pone.0020891 (PMC3137549; doi:10.1371/journal.pone.0020891)
Supplement: Supporting Information S1 — Stability of the disease-free and endemic equilibria is given in this file. (PDF) [file pone.0020891.s001.pdf]

Online Supporting Information

Analysis of Rabies in China: Transmission Dynamics and Control

Juan Zhang, Zhen Jin, Gui-Quan Sun, Tao Zhou, and Shigui Ruan

Stability of the disease-free equilibrium

By rewriting the variables of right side in model (1) into $x = \{E, I, E_1, I_1, S, R, S_1, R_1\}$, we can calculate that

$$\mathscr{F} = \begin{pmatrix} \beta SI \\ 0 \\ \beta_1 S_1 I \\ 0 \\ 0 \\ 0 \\ 0 \\ 0 \end{pmatrix}, \mathscr{V} = \begin{pmatrix} mE + \sigma(1-\gamma)E + kE + \sigma\gamma E \\ mI + \mu I - \sigma\gamma E \\ m_1E_1 + \sigma_1(1-\gamma_1)E_1 + k_1E_1 + \sigma_1\gamma_1E_1 \\ m_1I_1 + \mu_1I_1 - \sigma_1\gamma_1E_1 \\ mS + \beta SI + kS - [A + \lambda R + \sigma(1-\gamma)E] \\ mR + \lambda R - k(S+E) \\ m_1S_1 + \beta_1S_1I - [B + \lambda_1R_1 + \sigma_1(1-\gamma_1)E_1] \\ m_1R_1 + \lambda_1R_1 - k_1E_1 \end{pmatrix},$$

$$\mathscr{V}^- = \begin{pmatrix} mE + \sigma(1-\gamma)E + kE + \sigma\gamma E \\ mI + \mu I \\ m_1E_1 + \sigma_1(1-\gamma_1)E_1 + k_1E_1 + \sigma_1\gamma_1E_1 \\ m_1I_1 + \mu_1I_1 \\ mS + \beta SI + kS \\ mR + \lambda R \\ m_1S_1 + \beta_1)S_1I \\ m_1R_1 + \lambda_1R_1 \end{pmatrix}, \mathscr{V}^+ = \begin{pmatrix} 0 \\ \sigma\gamma E \\ 0 \\ \sigma_1\gamma_1E_1 \\ A + \lambda R + \sigma(1-\gamma)E \\ k(S+E) \\ B + \lambda_1R_1 + \sigma_1(1-\gamma_1)E_1 \\ k_1E_1 \end{pmatrix}.$$

$$J|_{E_0} = \begin{pmatrix} A & B \\ C & D \end{pmatrix},$$

where

$$A = \begin{pmatrix} -m-k & 0 & \sigma(1-r) & 0 \\ 0 & -m_1 & 0 & \sigma_1(1-r_1) \\ 0 & 0 & -m-\sigma-k & 0 \\ 0 & 0 & 0 & -m_1-\sigma_1-k_1 \end{pmatrix}, B = \begin{pmatrix} -\beta S^0 & 0 & \lambda & 0 \\ -\beta_1 S_1^0 & 0 & 0 & \lambda_1 \\ \beta S^0 & 0 & 0 & 0 \\ \beta_1 S_1^0 & 0 & 0 & 0 \end{pmatrix},$$

$$C = \begin{pmatrix} 0 & 0 & \sigma r & 0 \\ 0 & 0 & 0 & \sigma_1 r_1 \\ k & 0 & k & 0 \\ 0 & 0 & 0 & k_1 \end{pmatrix}, D = \begin{pmatrix} -m-\mu & 0 & 0 & 0 \\ 0 & -m_1-\mu_1 & 0 & 0 \\ 0 & 0 & -m-\lambda & 0 \\ 0 & 0 & 0 & -m_1-\lambda_1 \end{pmatrix}.$$

The five assumptions required in Theorem 2 of [1] can easily be satisfied by observing $\mathscr{F}, \mathscr{V}, \mathscr{V}^+, \mathscr{V}^-$ and $J|_{E_0}$. So, we can directly obtain the local asymptotical stable of E_0.

To prove its global attraction, notice that the last four equations are independent of the first four equations, we start by considering the first four equations.

$$\begin{cases} \frac{dS}{dt} = A + \lambda R + \sigma(1-\gamma)E - mS - \beta SI - kS, \\ \frac{dE}{dt} = \beta SI - mE - \sigma(1-\gamma)E - kE - \sigma\gamma E, \\ \frac{dI}{dt} = \sigma\gamma E - mI - \mu I, \\ \frac{dR}{dt} = k(S+E) - mR - \lambda R. \end{cases} \quad (1)$$

Because $R_0 < 1$, there is a small enough positive number ϵ such that $R_0 + \frac{\beta\epsilon\sigma\gamma}{(m+\mu)(m+\sigma+k)} < 1$. Now we prove that for ϵ, there is t_2 such that for all $t > t_2$, $S \leq S^0 + \epsilon$. From the last equation of Eqs.(4), we have

$$\begin{aligned}\frac{dR}{dt} &= k(S+E) - mR - \lambda R \\ &= k(N-R-I) - mR - \lambda R \\ &\leq k\frac{A}{m} - (k+m+\lambda)R.\end{aligned}$$

So we have that for $\epsilon_1 = \frac{\epsilon(m+k)}{\lambda}$, there is $t_1 > 0$ such that for all $t > t_1$, $R \leq \frac{kA}{m(m+\lambda+k)} + \epsilon_1 = R^0 + \epsilon_1$. Also from the first two equations of Eqs.(4), we have for all $t > t_1$ that

$$\begin{aligned}\frac{d(S+E)}{dt} &= A + \lambda R - m(S+E) - k(S+E) - \sigma\gamma E \\ &\leq A + \lambda(R^0+\epsilon_1) - (m+k)(S+E),\end{aligned}$$

then,

$$\lim_{t\to\infty}\sup(S+E) \leq \frac{A+\lambda(R^0+\epsilon_1)}{m+k}.$$

Because $E \geq 0$, it follows that

$$\begin{aligned}\lim_{t\to\infty}\sup S &\leq \frac{A+\lambda(R^0+\epsilon_1)}{m+k} \\ &= \frac{A+\lambda(\frac{A}{m}-S^0+\epsilon_1)}{m+k} \\ &= S^0 + \frac{\lambda\epsilon_1}{m+k}.\end{aligned}$$

Thus, there is t_2 such that for all $t > t_2 > t_1$, $S \leq S^0 + \epsilon$, where $\epsilon = \frac{\lambda\epsilon_1}{m+k}$.

When $t > t_2 > t_1$, we consider the following system:

$$\begin{cases}\frac{dE}{dt} \leq \beta(S^0+\theta)I - mE - \sigma(1-\gamma)E - kE - \sigma\gamma E, \\ \frac{dI}{dt} = \sigma\gamma E - mI - \mu I.\end{cases} \tag{2}$$

From the corresponding linear system

$$\begin{cases}\frac{d\hat{E}}{dt} = \beta(S^0+\epsilon)\hat{I} - m\hat{E} - \sigma(1-\gamma)\hat{E} - k\hat{E} - \sigma\gamma\hat{E}, \\ \frac{d\hat{I}}{dt} = \sigma\gamma\hat{E} - m\hat{I} - \mu\hat{I},\end{cases} \tag{3}$$

we obtain its characteristic equation:

$$W^2 + (2m+k+\mu+\sigma)W + (m+k+\sigma)(m+\mu) - \beta(S^0+\epsilon)\sigma r = 0.$$

Since $R_0 < 1$ and $\epsilon > 0$ is sufficiently small, we have

$$(m+k+\sigma)(m+\mu) - \beta(S^0+\epsilon)\sigma r > 0.$$

Thus, the eigenvalues ω_1 and ω_2 of the linear system are negative. It follows that the solution of the system (5)

$$x(t) = c_1 p_1 e^{\omega_1 t} + c_2 p_2 e^{\omega_1 t} \to 0,\ t \to \infty,$$

where p_1 and p_2 are the corresponding eigenvectors of the eigenvalues ω_1 and ω_2, respectively, and c_1, c_2 are arbitrary constants. Applying the comparison principle [2], we get that $E(t) \to 0$ and $I(t) \to 0$ as $t \to \infty$. By the theory of asymptotic autonomous systems [3], it is known that $S(t) \to S^0, R(t) \to R^0$ as $t \to \infty$. So E_0 is globally attractive when $R_0 < 1$.

Next, we consider the last four equations:

$$\begin{cases} \frac{dS_1}{dt} = B + \lambda_1 R_1 + \sigma_1(1-\gamma_1)E_1 - m_1 S_1 - \beta_1 S_1 I, \\ \frac{dE_1}{dt} = \beta_1 S_1 I - m_1 E_1 - \sigma_1(1-\gamma_1)E_1 - k_1 E_1 - \sigma_1\gamma_1 E_1, \\ \frac{dI_1}{dt} = \sigma_1\gamma_1 E_1 - m_1 I_1 - \mu_1 I_1, \\ \frac{dR_1}{dt} = k_1 E_1 - m_1 R_1 - \lambda_1 R_1. \end{cases} \tag{4}$$

Because when $t \to \infty, I \to 0$, the limiting system of Eqs.(6) is $\frac{dS_1}{dt} = B - m_1 S_1$, whose disease-free equilibrium is $(\frac{B}{m_1}, 0, 0, 0)$ which is globally asymptotically stable. According to the theory of asymptotic autonomous systems, Theorem 1.2 in [3], $(\frac{B}{m_1}, 0, 0, 0)$ is also the disease-free equilibrium of Eqs.(6) and is globally asymptotically stable. Thus, the disease-free equilibrium E_0 is globally asymptotically stable in the region Γ when $R_0 < 1$.

Local stability of the endemic equilibrium

It is obvious that the disease-free equilibrium E_0 is an attractor in $\{(S, E, I, R, S_1, E_1, I_1, R_1) \in \Gamma : I_1 = 0\}$. The Jacobian matrix of system (1) at the endemic equilibrium E_* is

$$J|_{E_*} = \begin{pmatrix} A & B \\ C & D \end{pmatrix},$$

where

$$A = \begin{pmatrix} -m-\beta I^*-k & 0 & \sigma(1-r) & 0 \\ 0 & -m_1-\beta_1 I^* & 0 & \sigma_1(1-r_1) \\ \beta I^* & 0 & -m-\sigma-k & 0 \\ 0 & \beta_1 I^* & 0 & -m_1-\sigma_1-k_1 \end{pmatrix},$$

$$B = \begin{pmatrix} -\beta S^* & 0 & \lambda & 0 \\ -\beta_1 S_1^* & 0 & 0 & \lambda_1 \\ \beta S^* & 0 & 0 & 0 \\ \beta_1 S_1^* & 0 & 0 & 0 \end{pmatrix}, C = \begin{pmatrix} 0 & 0 & \sigma r & 0 \\ 0 & 0 & 0 & \sigma_1 r_1 \\ k & 0 & k & 0 \\ 0 & 0 & 0 & k_1 \end{pmatrix},$$

and

$$D = \begin{pmatrix} -m-\mu & 0 & 0 & 0 \\ 0 & -m_1-\mu_1 & 0 & 0 \\ 0 & 0 & -m-\lambda & 0 \\ 0 & 0 & 0 & -m_1-\lambda_1 \end{pmatrix}.$$

Through calculating, we derive the characteristic polynomial

$$\begin{aligned} P(x) \;=\; & (x+m_1+\mu_1)[x^3 + (a+c+m_1+\beta_1 I^*)x^2 + (ac+am_1+cm_1+\beta_1 I^* b+\beta_1 I^* c)x \\ & +cm_1 a + (bc-\lambda_1 k_1)\beta_1 I^*]\{x^4 + (a_1+b_1+c_1+d_1+\beta I^*)x^3 + [c_1 d_1 + \beta I^* d_1 + \sigma r \beta S^* \\ & +(a_1+b_1)(d_1+c_1+\beta I^*) + (a_1 b_1 - \lambda k) + \sigma r \beta I^*]x^2 + [(a_1+b_1)(\beta I^* d_1 + \sigma r \beta S^*) \\ & +(a_1 b_1 - \lambda k)(d_1+c_1+\beta I^*) + \sigma r \beta I^*(b_1+d_1)]x + (a_1 b_1 - \lambda k)(c_1 d_1 + \beta I^* d_1 + \sigma r \beta S^*) \\ & +\sigma r \beta I^* b_1 d_1\}, \end{aligned}$$

where $a = m_1+\sigma_1+k_1, b = m_1+\sigma_1 r_1+k_1, c = m_1+\lambda_1, a_1 = m+k, b_1 = m+\lambda, c_1 = m+\sigma+k, d_1 = m+\mu$. Apparently, $-(m_1 + \mu_1)$ is a root of $P(x)$. Moreover, we consider $P_1(x) = x^3 + Ax^2 + Bx + C$, where,

$$\begin{aligned} A &= a + c + m_1 + \beta_1 I^*, \\ B &= ac + am_1 + cm_1 + \beta_1 I^* b + \beta_1 I^* c, \\ C &= cm_1 a + (bc - \lambda_1 k_1)\beta_1 I^*. \end{aligned}$$

It follows that

$$\begin{aligned} A &= (a + c + m_1 + \beta_1 I^*) > 0, \\ AB - C &= (a + c + m_1 + \beta_1 I^*)(ac + am_1 + cm_1 + \beta_1 I^* b + \beta_1 I^* c) \\ &\quad -[cm_1 a + (bc - \lambda_1 k_1)\beta_1 I^*] > 0, \\ C &= cm_1 a + (bc - \lambda_1 k_1)\beta_1 I^* > 0. \end{aligned}$$

By the Routh-Hurwitz criterion, we know that all roots of $P_1(x)$ have the negative real parts.
Further, we consider the polynomial

$$P_2(x) = x^4 + A_1 x^3 + B_1 x^2 + C_1 x + D_1,$$

where

$$\begin{aligned} A_1 &= a_1 + b_1 + c_1 + d_1 + \beta I^*, \\ B_1 &= c_1 d_1 + \beta I^* d_1 + \sigma r \beta S^* + (a_1 + b_1)(d_1 + c_1 + \beta I^*) + (a_1 b_1 - \lambda k) + \sigma r \beta I^*, \\ C_1 &= (a_1 + b_1)(\beta I^* d_1 + \sigma r \beta S^*) + (a_1 b_1 - \lambda k)(d_1 + c_1 + \beta I^*) + \sigma r \beta I^*(b_1 + d_1), \\ D_1 &= (a_1 b_1 - \lambda k)(c_1 d_1 + \beta I^* d_1 + \sigma r \beta S^*) + \sigma r \beta I^* b_1 d_1. \end{aligned}$$

It follows that

$$\begin{aligned} A_1 &= a_1 + b_1 + c_1 + d_1 + \beta I^* > 0, \\ D_1 &= (a_1 b_1 - \lambda k)(c_1 d_1 + \beta I^* d_1 + \sigma r \beta S^*) + \sigma r \beta I^* b_1 d_1 > 0, \\ A_1 B_1 - C_1 &= (a1 + b1 + c1 + d1 + \beta I^*)[c_1 d_1 + \beta I^* d_1 + \sigma r \beta S^* + (a_1 + b_1)(d_1 + c_1 + \beta I^*) \\ &\quad +(a_1 b_1 - \lambda k) + \sigma r \beta I^*] - [(a_1 + b_1)(\beta I^* d_1 + \sigma r \beta S^*) + (a_1 b_1 - \lambda k)(d_1 + c_1 + \beta I^*) \\ &\quad +\sigma r \beta I^*(b_1 + d_1)] > 0, \\ E &= C_1(A_1 B_1 - C_1) - D_1(A_1)^2 > 0. \end{aligned}$$

The first three inequalities are apparent from the formulas. However, the last one is not easy to calculate. We verified it by using MATLAB. Because the formula is too long, we do not list it here. By the Routh-Hurwitz criterion, all roots of $P_2(x)$ have the negative real parts. Therefore, the characteristic roots of $J|_{E_*}$ have negative real parts.

References

1. Diekmann O, Heesterbeek JAP, Roberts MG (2010) The construction of next-generation matrices for compartmental epidemic models. *J Royal Soc Interface* 7: 873-885.

2. Smith HL, Waltman P (1995) *The Theory of the Chemostat.* Cambridge University Press.

3. Thieme HR (1992) Convergence results and a Poincaré-Bendixson trichotomy for asymptotically automous differential equations. *J Math Biol* 30: 755-763.
